# The Allergic Rhinitis Clinical Investigator Collaborative (AR-CIC): verification of nasal allergen challenge procedures in a study utilizing an investigational immunotherapy for cat allergy

**DOI:** 10.1186/s13601-018-0198-7

**Published:** 2018-04-12

**Authors:** Helen Neighbour, Mena Soliman, Lisa M. Steacy, Pascal Hickey, Beth Forbes, Mark Larché, Anne K. Ellis

**Affiliations:** 1 Divisions of Clinical Immunology & Allergy and Respirology, Department of Medicine, Firestone Institute of Respiratory Health, The Research Institute, St. Joe’s Hamilton and McMaster University, Hamilton, ON Canada; 2 Allergy Research Unit, Kingston General Hospital, Kingston, ON Canada; 3 Adiga Life Sciences, Inc, McMaster Innovation Park, Hamilton, ON Canada; 4 Division of Allergy and Immunology, Department of Medicine, Kingston General Hospital, Queen’s University, 76 Stuart Street, Watkins D1, Kingston, ON K7L 2V7 Canada

**Keywords:** Allergic Rhinitis, Nasal allergen challenge, Immunotherapy, Peptide, Cat allergy, Epitope

## Abstract

**Background:**

The Allergic Rhinitis Clinical Investigator Collaborative (AR-CIC) is a network of experienced Allergic Rhinitis (AR) researchers developing better research tools based on the nasal allergen challenge (NAC). A key objective of such is the ability to detect efficacy in a small population. AR-CIC sought to test its NAC protocol as a secondary objective in two small mechanistic research trials of a novel form of immunotherapy [Cat Peptide Antigen Desensitisation (Cat-PAD)] for which efficacy had previously been demonstrated. The primary objective (not presented here) was to identify potential biomarkers of efficacy for peptide immunotherapy, and this provided an ideal opportunity to corroborate the NAC protocol. We aim to clinically validate the AR-CIC NAC methodology in a pooled analysis of secondary endpoints measured in two open label mechanistic studies of cat allergic participants treated with Cat-PAD.

**Methods:**

Cat allergic AR sufferers with ongoing cat exposure were included. Participants had to demonstrate a total nasal symptom score (TNSS) of at least 8 (max 12) and/or achieve a reduction in peak nasal inspiratory flow (PNIF) of ≥ 50% during a screening titrated NAC. Eligible participants then underwent a baseline NAC visit with the allergen dose that produced a positive challenge at screening, followed by four monthly injections of 6 nmol Cat-PAD. A follow up NAC visit documented changes in nasal response 1 month following the completion of treatment.

**Results:**

Nineteen subjects completed the study protocol in the two studies combined. Four injections of Cat-PAD resulted in a significant reduction in TNSS responses generated via NAC following allergen challenge (15 min *p* < 0.05, 30 min *p* < 0.05, 1 h *p* < 0.01, 2 h *p* < 0.05). There was modest correlation between symptom scores and PNIF measurements.

**Conclusions:**

This study supports the validity of the AR-CIC’s optimised NAC protocol for conducting research of the potential efficacy of novel therapeutics in multi-centre studies.

*Trial registration* Both studies reported herein were registered clinicaltrials.gov (NCT01383590 and NCT01383603)

## Background

Allergic Rhinitis (AR) is an inflammatory disease of the nasal mucosa, manifesting in symptoms of rhinorrhea, sneezing, nasal congestion and itch as a result of exposure to specific allergens [1]. According to the Allergic Rhinitis and its Impact on Asthma (ARIA) report, it is conservatively estimated that five hundred million people suffer from AR globally [1]. Disease burden is usually manifest by fatigue, poor concentration, and reduced productivity, in addition to worsening of associated diseases such as allergic asthma [2].

Nasal Allergen Challenge (NAC) protocols have been developed for the purpose of studying the efficacy of novel medications for AR [3–5]. Different variations of this protocol have been used, however, all entail the direct exposure of the nasal mucosa to the allergen of interest through various methods. Participants can then evaluate their symptoms, such as nasal congestion, rhinorrhea, and sneezing, and a variety of objective measures can be completed such as peak nasal inspiratory flow (PNIF) or acoustic rhinometry [3]. Baseline measurements are later compared to recordings following the allergen challenge, at pre-set intervals. By repeating the challenge once more following a course of the investigational treatment, a comparison of both subjective and objective measures pre- and post-treatment can help evaluate the performance of the novel therapeutic [3]. Biological samples may also be collected during these studies to gain a better understanding of the mechanistic action of the therapeutic under study. Studying the effect of therapy on eosinophil counts in blood and nasal lavage, in addition to a variety of nasal cytokines at specific time points, can provide clues to the mechanistic pathways effected by therapy [6–8]. Specific gene expression analysis, particularly of those genes encoding pro-inflammatory cytokines might also provide valuable information about the effects of treatment [9, 10].

The Allergic Rhinitis—Clinical Investigator Collaborative (AR-CIC), part of the Allergy, Genes and the Environment Networks for Centres of Excellence (AllerGen NCE), has developed a NAC protocol for evaluating novel AR therapies clinically and to aid in understanding their mechanism of action [4]. The AR-CIC’s optimized protocol enables the frequent monitoring of AR symptoms and nasal airflow in addition to collection of a variety of biologic samples. Samples such as blood (serum, plasma, peripheral blood mononuclear cells), Rhinoprobe™ (Arlington Scientific, UT, USA) tissue samples for studying changes in gene expression or epigenetic modifications, synthetic absorptive matrices (SAMs) for evaluation of secretions for nasal cytokines, and nasal lavage samples for studying nasal cellular populations, enable better understanding of the mechanism of action of a novel therapy [3]. A variety of similar techniques were employed in the past to test novel therapies for AR using different ways for allergen challenge and sample collection [11–14]. The rationale behind this study, which comprises a pooled analysis of two clinical trials conducted at two separate Canadian sites, was to demonstrate the utility of the AR-CIC protocol for detecting signals of efficacy for a novel immunotherapeutic agent in a relatively small population of 20 subjects.

Cat peptide antigen desensitisation (Cat-PAD, Circassia Ltd, Oxford, UK) is an equimolar mixture of seven short synthetic peptides currently under development for the treatment of allergies to cat dander [15–17]. The peptide sequences range from 13 to 17 amino acids in length and are derived from the primary sequence of the major cat allergen Fel d 1. Cat-PAD is designed to activate allergen specific T-cells in the absence of simultaneous inflammatory triggers (i.e. those resulting from the cross-linking of IgE on mast cells and basophils) to induce immune tolerance over a relatively short, standardised treatment course.

The clinical assessment of novel immunotherapies such as Cat-PAD is challenging. Variability in a subject’s exposure to allergen under real life conditions results in inconsistent frequency and magnitude of allergic symptoms, which means that large clinical efficacy trials are likely to be required to achieve statistical power. Ideally, before embarking on large trials, which are expensive and time consuming, “proof of concept” would be obtained in smaller, focused studies. However, there are a limited number of models for demonstrating proof of concept in allergy and thus a need exists for additional tools to evaluate in an efficient manner the potential efficacy of new immunotherapies. Such proof-of-concept studies would ideally also provide important information on mechanism of action at an early stage of drug development. Nasal allergen challenge models have been utilized but differences certainly exist in the literature, and the AR-CIC have aimed to capture the strengths noted over their own past experience but also integrating novel approaches to optimize a standardized protocol that can be used in future clinical trials.

We hereby report that the AR-CIC’s optimised NAC protocol was able to detect significant changes in both subjective and objective measurements of clinical efficacy, in an open label clinical trial of the novel immunotherapeutic Cat-PAD utilizing a population of only 20 allergic subjects.

## Methods

The AR-CIC NAC protocol was included in two small, open-label mechanistic research studies which each had the primary objective of seeking to identify potential biomarkers of Cat-PAD efficacy (RES-003, Kingston General Hospital, ON, Canada and RES-004, St. Josephs Healthcare Hamilton, ON, Canada). The only difference between the two studies (and also the rationale for conducting the evaluations under two separate protocols) was that the blood sampling regime in RES-003 was designed for proteomic investigations in whole blood, whereas the blood sampling regime in RES-004 was designed to enable transcriptomic analyses within Fel d 1-specific T cells. To enable the isolation of specific T cells, RES-004 included an additional inclusion criterion to ensure only subjects with certain tissue types were enrolled into the study. The trials were approved by Health Canada and the Research Ethics Board’s (REB) at each site, were conducted in accordance with the Declaration of Helsinki and were registered at clinicaltrials.gov (NCT01383590 and NCT01383603). Written informed consent was obtained from all participants prior to the conduct of any study related visits.

These studies were open label, single centre studies using the proposed clinical regimen of Cat-PAD. Secondary endpoints in each study included measurement of changes in NAC-induced nasal symptoms and inspiratory flow following treatment, with the intention of subsequently pooling the NAC results from the two trials in order to achieve a subject population of 20 which was considered sufficient to address AR-CIC’s objective of validating the use of the NAC protocol for the study of novel therapeutics. Other biological samples were collected and included nasal lavage for eosinophil cell counts, nasal cytokine samples using SAMs, nasal epithelial cells samples using Rhinoprobes^®^, in addition to various blood samples at pre-specified time points.

**Fig. 1:**
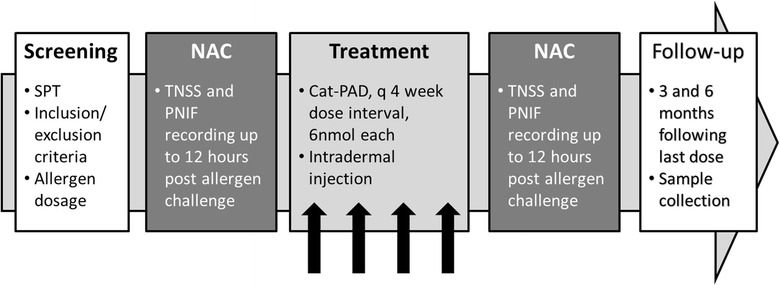
Study overview. A total of 19 participants completed the study

A schematic overview of clinical trial design with respect to the AR-CIC objective is presented in Fig. 1. In total, 20 participants, between the ages of 18–65 years of age, were enrolled at Queen’s and McMaster Universities (Ontario, Canada), of which 19 participants completed the study.

During the initial screening visit, a medical history, physical exam, measurement of the forced expiratory volume in the first second (FEV1), routine blood haematology and biochemistry, and urine analyses were conducted to determine eligibility. Participants had to have a minimum of 1 year of documented history of AR on exposure to cats, and a positive skin prick test to cat pelt allergen (wheal diameter ≥ 3 mm larger than the negative control, ALK Abello). Participants were required to have regular exposure to a cat in their normal living or working environment throughout the study period, with regular exposure defined as exposure to a cat or to an environment that normally contains cats, on at least two occasions and for a duration of at least 8 h per week.

Potential participants were excluded if they were diagnosed with asthma, however, occasional wheeze or a diagnosis of exercise induced bronchospasm were not grounds for exclusion. An FEV1 of < 80% of predicted, or an FEV1/Forced Vital Capacity (FVC) of < 70% were grounds for exclusion at screening (Table 1). A history of anaphylaxis to cat allergen, receipt of any allergen immunotherapy in the last 10 years, or receipt of non-adjuvant or non-sublingual pre-seasonal immunotherapy in the last 3 years, were also exclusion criteria. Please refer to Table 1 for a full list of exclusion criteria and washout periods for medications.

**Table 1:** Inclusion and exclusion criteria, including required washouts for restricted medications

Inclusion criteria	
Male or female aged 18–65 years	Willing and able to provide written consent
Minimum 1-year documented history of AR on exposure to cats	Willing and able to participate in all study visits, treatment plans, and provide all samples
Positive SPT to cat allergen ≥ 3 mm than negative control	TNSS ≥ 8/12 or PNIF reduction ≥ 50% on screening allergen challenge
Regular exposure to a cat in their normal living or working environment, on at least 2 separate occasions for a total duration of at least 8 h per week	Participants of childbearing age must practice an acceptable form of contraception and have a negative urine pregnancy test at screening

Exclusion criteria	
Diagnosis of asthma	Clinically relevant physical examination abnormalities (in the investigator’s opinion)
Laboratory values outside of normal limits except if deemed not of clinical relevance by the investigator	Vital signs outside of normal limits, except if deemed not of clinical relevance by the investigator
FEV1 < 80% of predicted	FEV1/FVC < 70%
History of anaphylaxis	Significant history of alcohol and drug abuse
Receipt of any allergen immunotherapy within the last 10 years, or in the last 3 years for non-adjuvant, or non-sublingual pre-seasonal immunotherapy treatments	History of any significant disease or disorder such as autoimmune, cardiovascular, pulmonary, which in the opinion of the investigator would either put the participant at risk, or affect the study results, or the participant’s ability to take part in the study
If epinephrine administration is contra-indicated	History of vaso-vagal reaction in response to needles and blood donation
Clinically relevant illness, in the investigator’s opinion, within the previous 6 weeks	Previous participation in this or previous study of Cat-PAD
Participants who are pregnant, lactating, or planning a pregnancy	History of severe drug allergy, severe angioedema, or anaphylactic reaction to food
Received treatment with an investigational drug within 3 months	Unable to communicate or to understand the requirements of the study
Known allergy to thioglycerol	History of immune-pathological diseases
History of positive test results to Hepatitis B, Hepatitis C, HIV, or tuberculosis other than anticipated due to vaccination	Smoker or quit smoking in the past 3 months
In the opinion of the investigator, the participant is unlikely to complete all of the study	Must abide with washout periods to certain medications

Medication	Washout period prior to Screening Visit and NAC Visits
Depot corticosteroids	90 days
Tricyclic antidepressant	14 days
Monoamine oxidase inhibitors (MAOI)	14 days
Beta-blockers	Investigator’s decision
Alpha-adrenoceptor blocker	Investigator’s decision
Tranquillizers or psychoactive drugs, except benzodiazepines and zopiclone on a PRN basis (no more than twice per month)	14 days
Treatment of minor affective disorders with stable doses of non-tricyclic anti-depressants such as serotonin antagonists and reuptake inhibitors, selective serotonin reuptake inhibitors, serotonin norepinephrine reuptake inhibitors and norepinephrine-dopamine reuptake inhibitors	Dose administered must remain constant from 6 weeks prior to enrolment
Corticosteroids (systemic, dermatological*, inhalational, intranasal, ocular)	30 days
Anti-histamines (nasal, long acting) once daily dosing oral	10 days
Anti-histamines (nasal, short acting) more than once daily dosing, oral or ocular	7 days
Anticholinergics	7 days
Alpha-adrenergic agonists	7 days
Cromones	30 days
Leukotriene inhibitors	10 days
Non-steroidal anti-inflammatory drugs (NSAIDs)	3 days prior to NAC

* Topical hydrocortisone (≤ 1%) was permitted for use on < 10% body surface area

**Fig. 2:**
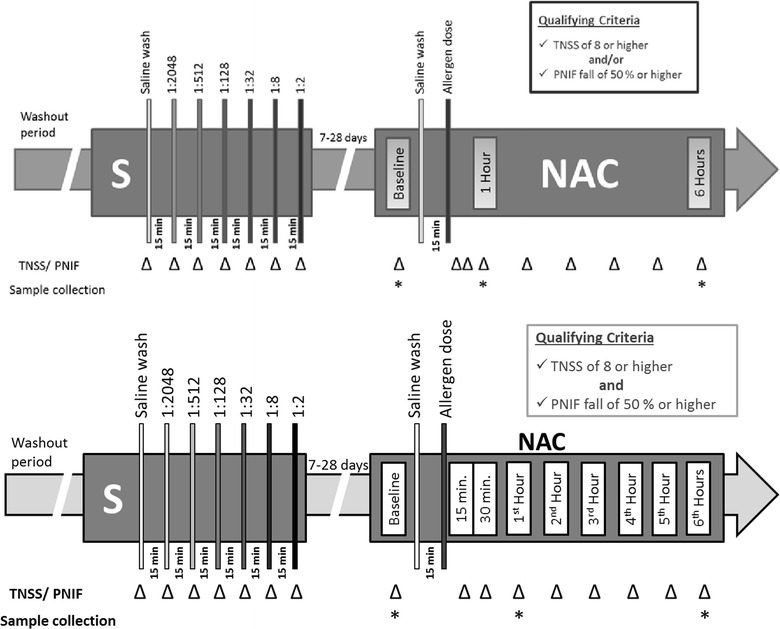
TNSS and PNIF data were obtained at baseline, 15, 30 min, 1 h and then hourly for a minimum of 6 h during each NAC visit. Once baseline measurements were recorded, participants received a saline wash of the nasal cavity and were challenged with the qualifying dose of cat dander extract determined during the screening visit

If all criteria were met, the nasal cavity was washed with saline and participants recorded their nasal symptoms of congestion, sneezing, rhinorrhea, and nasal itchiness, each graded from 0 to 3, and these scores were combined to form the Total Nasal Symptom Score (TNSS) which had a possible total score of 12. Participants who developed a TNSS of > 2 following the saline wash were excluded. Peak Nasal Inspiratory Flow (PNIF) was also recorded using a hand held nasal mask and meter (InCheck, Clement Clarke International Ltd). Participants were challenged intra-nasally with escalating (fourfold) increments of standardized cat dander extract (ALK-Abello), at 15 min intervals, until a TNSS of at least 8 and/or a PNIF reduction of ≥ 50% were reached (Fig. 2). This screening NAC procedure was performed to confirm eligibility and establish the allergen challenge dose that would be used at subsequent baseline and post-treatment NAC visits (Table 2).

**Table 2:** Allergen concentrations at screening and corresponding cumulative concentration received during NAC

Screening challenge dilution	Actual (cumulative) dose delivered at NAC Visit
1:128	1:128
1:32	1:24
1:8	1:6
1:2	1:2

Seven to 28 days following the screening visit, the baseline (i.e. pre-treatment) NAC visit was conducted using the allergen challenge dose that was established at screening. TNSS and PNIF were recorded at baseline, 15, 30 min, 1 h, and hourly up to 6 h post-NAC on site.

The treatment visits commenced up to a maximum of 8 weeks following the pre-treatment NAC visit. At each of four treatment visits (scheduled 4 weeks apart), the participants’ exposure to cats was recorded, inclusion and exclusion criteria reviewed, and adverse events recorded before and after receiving an intradermal injection of 6 nmol Cat-PAD. Cat-PAD was supplied as a lyophilisate (Circassia Ltd, Oxford UK) to which water for injection was added for reconstitution, such that a final dosing volume of 60 μl contained 6 nmol of each of the peptides.

A follow-up NAC was conducted 1 month after the last dose of Cat-PAD using the same fixed allergen dose and sample timing as described above for the pre-treatment NAC. One more follow-up visit was conducted 6 months following administration of the last dose of Cat-PAD (Fig. 1). The purpose of the final follow up visits were to collect blood samples for biomarker analysis.

GraphPad Prism 6.0.7 (San Diego, California, USA) was used for statistical analysis of the data and generation of graphs. Repeated measures ANOVA with Bonferroni correction was used to compare pre and post treatment TNSS and PNIF. Wilcoxon signed rank test was used to compare overall TNSS and PNIF change between the NAC visits. Correlation analysis was calculated using Pearson’s correlation coefficient.

## Results

**Fig. 3:**
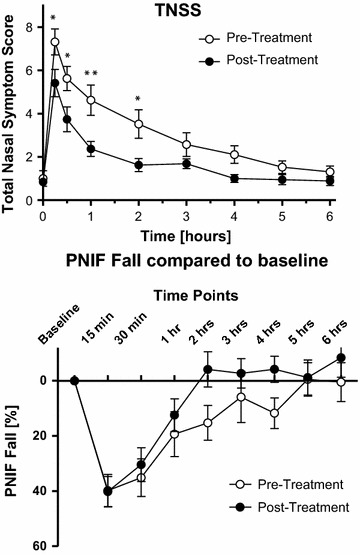
Effect of Cat-SPIRE on TNSS and PNIF. Participants (n = 19) experienced an initial peak in TNSS and a peak reduction in PNIF at 15 min following allergen challenge, followed by a gradual return to baseline values. Following treatment, participants experienced a significant reduction in TNSS at 15 min (*p* < 0.05), 30 min (*p* < 0.05), 1 h (*p* < 0.01), and 2 h (*p* < 0.05). Fall in PNIF in response to allergen challenge was also reduced after treatment though not evident in statistical analysis

A total of 54 participants were screened for the two studies. Six participants did not achieve the qualifying criteria of a TNSS of 8/12 and/or a PNIF reduction of 50% from baseline values and eight subjects were excluded from RES-004 because they did not have a matching tissue type required for mechanistic studies (refer Materials and Methods). The other 20 screened participants did not meet the remaining inclusion criteria. Four subjects were enrolled into the study at the Hamilton site on the basis of achieving either the TNSS or PNIF criterion (as opposed to both). One of the 20 enrolled subjects was not able to attend all of the scheduled visits following the pre-treatment NAC due to relocation to another city. Nineteen subjects therefore received the scheduled four doses of Cat-PAD and attended both the pre- and post-treatment NAC visits. At the pre-treatment NAC, participants experienced an increase in TNSS, reaching a mean peak score of 7.32 at 15 min following the allergen challenge (Fig. 3). This was followed by a gradual decline to values close to baseline by hour 6 following the allergen challenge. Mean PNIF was markedly reduced from a baseline of 121.75 to 67.75 L/min, a 44% reduction in PNIF, 15 min following the NAC. In a similar manner to TNSS, PNIF gradually returned to baseline values by hour 6.

During the post-treatment NAC, the TNSS and PNIF versus time profiles followed a similar pattern to the pre-treatment NAC, but lower symptom scores and reductions in airflow were recorded as compared to the measurements taken during pre-treatment NAC. Mean TNSS reached a peak of 5.42 at 15 min, significantly lower than peak TNSS during pre-treatment NAC 7.32 (*p* < 0.05), a 26% reduction in mean symptom scores. Mean TNSS was also significantly lower during post-treatment at 30 min (*p* < 0.05), 1 h (*p* < 0.01), and 2 h (*p* < 0.05), after which the TNSS for both pre and post-treatment NAC returned to near baseline values (Fig. 3). There was an overall significant change in TNSS after treatment (*p* < 0.01).

The reduction in nasal airflow (PNIF) following allergen challenge did not change significantly after treatment, although there was evidence of an effect, particularly at 2 h (99.3 L/min pre-treatment vs 120.5 L/min post-treatment) and at 4 h (103.7 L/min per-treatment vs 122.6 L/min post-treatment), a 17.6 and 15.5% improvement in airflow respectively.

**Fig. 4:**
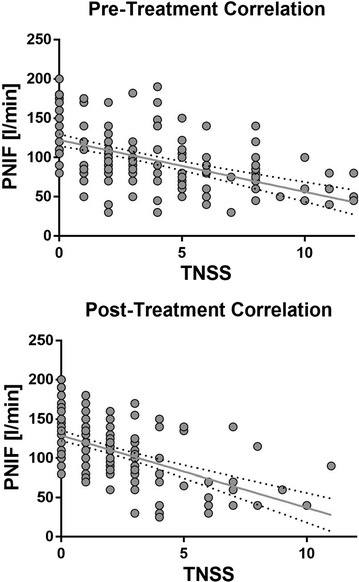
Modest correlation between TNSS and PNIF before and following treatment. Every TNSS/PNIF value recorded by each participant at each time point has been plotted separately. The variability in PNIF between participants has resulted in modest correlation (Pre-treatment: R^2^ = 0.257, *p* < 0.0001; post-treatment: R^2^ = 0.270, *p* < 0.0001). Significant correlation is observed between mean TNSS and mean PNIF across the time course of NAC for both pre-treatment (R^2^ = 0.941, *p* < 0.0001) and post-treatment (R^2^ = 0.95, *p* < 0.0001) NAC visits

Correlation between the objective PNIF and subjective TNSS evaluation methods based on the mean data for the 19 subjects at each time point was high both at the pre-treatment (r^2^ = 0.941, *p* < 0.0001) and post-treatment (r^2^ = 0.95, *p* < 0.0001) NAC visits using Pearson’s correlation coefficient (Fig. 4). Plotting all individual TNSS values against corresponding PNIF values measured at the same time point resulted in modest correlation (Pre-treatment: R^2^ = 0.257, *p* < 0.0001; post-treatment: R^2^ = 0.270, *p* < 0.0001), owing primarily to the variability in PNIF values, but a general trend was still observed with PNIF decreasing as TNSS increases (Fig. 4). Post-treatment analysis showed greater clustering of points to the left of the graph compared to more widespread distribution prior to treatment.

Nineteen participants experienced at least one treatment emergent adverse event (TEAE). Where information was available, the majority of the events were indicated as mild or moderate in intensity with three subjects experiencing five severe TEAEs, all of which were considered unrelated to treatment. None of these events were serious and none led to withdrawal from the study. The majority of the TEAEs were related to infections, including upper respiratory tract infection, gastrointestinal viral infection, influenza, nasopharyngitis, and urinary tract infection.

There were six events of hypersensitivity in 5/19 (26%) subjects. One subject experienced an adverse event of allergies to an unknown trigger of moderate severity that occurred 3 months after the last dose of Cat-PAD. It was considered unrelated to treatment. Another subject experienced worsening of allergic symptoms 22 days after dose 3 and again 2 days after dose 4, however, neither were considered treatment related. The remaining 3 events all occurred 8 days or more after a dose of study medication. One that occurred 8 days after dose 4 was judged possibly related, one that occurred 11 days after dose 2 was considered not related and a causality report was not available for the last event which occurred 1 month after the fourth dose. One subject experienced two episodes of urticaria which were both of moderate severity, both more than 5 months after the last dose of Cat-PAD and both judged to be unrelated. One subject experienced mild pruritis with dosing which lasted for 1 h and considered related and resolved without treatment.

Three subjects had respiratory system adverse events. One of these subjects, with no history of asthma, experienced mild related events of allergic cough, dyspnea and chest discomfort 7 days after the first dose. These events resolved without treatment and did not recur with subsequent doses. Another subject with a past history of respiratory wheeze, experienced chest discomfort and wheezing on the day of the third dose. The event was considered related and resolved without treatment and did not recur following the final dose. The final subject had a cough which started 30 days after the fourth dose and was considered possibly related. There were no clinically relevant effects of either the study procedures or treatment with Cat-PAD on peak expiratory flow rate or FEV1.

Injection site reactions were reported post-dose in the majority of subjects across the dosing visits in both studies with no reactions seen at Follow-up. These were categorized as mild or not bothersome.

## Discussion

Cat-PAD, a novel synthetic peptide immunotherapy, has been previously shown to be effective in reducing AR symptoms in placebo-controlled phase IIB clinical trials [18, 19]. It was effective in attenuating AR symptoms during an evaluation in a Controlled Allergen Challenge Facility (CACF) 1 year after the start of treatment with a single treatment regimen of four intradermal injections of a 6 nmol, with evidence of sustained effects after 2 years following dose [18]. The primary mechanistic objective of these two clinical studies was to identify proteomic and gene transcriptomic biomarkers of Cat-PAD treatment. The proteomic and transcriptomic studies are currently underway and will be reported elsewhere when complete. It is important to emphasize that the objective of this study was not to serve as proof of efficacy for Cat-PAD, but rather one that validated the NAC protocol for use in clinical trials.

The AR-CIC’s NAC protocol was previously optimized in multi-centre studies using a variety of allergens [4]. The protocol was shown to reliably produce objective measurements and subjective symptoms following an allergen challenge [4]. The TNSS and PNIF patterns observed following the challenging of participants with cat dander extract were similar to that observed in the AR-CIC’s earlier studies, where most participants reached their maximum TNSS 15 min following the allergen challenge followed by a gradual decline [4].

Earlier studies have demonstrated that NAC procedures produced consistent results when separated by intervals of at least 4 weeks [20], thus providing a robust tool for assessing the effects of a short course of immunotherapy. The significant reduction in NAC-induced TNSS observed in this study suggests that the model was able to detect clinical efficacy of a therapeutic AR agent in a relatively small population, but as it was open-label and there was no placebo arm, no firm conclusions of efficacy can be drawn. However, the symptom score data may provide significant value in the interpretation of proteomic and transcriptomic data generated from the same subject population [3, 21, 22].

The correlation between the subjective TNSS and objective PNIF measurements, while using cat allergen extract, was a similar finding to other AR-CIC studies using ragweed allergen [4]. The correlation between these two assessments serves to strengthen the confidence and utility of measuring both subjective and objective endpoints, though individual points provided modest results compared to mean values. Certainly subjective endpoints in the absence of objective ones are considered scientifically weak, whereas objective endpoints in the absence of subjective ones raise questions about relevance to clinical practice and patient outcomes.

## Conclusions

This study supports the validity of the AR-CIC’s optimised NAC protocol for conducting research of the potential efficacy of novel therapeutics in multi-centre studies.

## Abbreviations

AR: Allergic Rhinitis
AR-CIC: Allergic Rhinitis Clinical Investigator Collaborative
ARIA: Allergic Rhinitis and its impact on asthma
Cat PAD: Cat Peptide Antigen Desensitisation
FEV1: forced expiratory volume in the first second
FVC: Forced Vital Capacity
PNIF: peak nasal inspiratory flow
NAC: nasal allergen challenge
TNSS: total nasal symptom score
